# Financial burden of men with localized prostate cancer: a process paper

**DOI:** 10.3389/fpsyg.2023.1176843

**Published:** 2023-07-05

**Authors:** Ashley J. Housten, Hannah E. Rice, Su-Hsin Chang, Allison J. L'Hotta, Eric H. Kim, Bettina F. Drake, Robin Wright-Jones, Mary C. Politi

**Affiliations:** ^1^Division of Public Health Sciences, Department of Surgery, Washington University School of Medicine, St. Louis, MO, United States; ^2^Division of Urology, Department of Surgery, Washington University School of Medicine, St. Louis, MO, United States; ^3^The Empowerment Network, St. Louis, MO, United States

**Keywords:** prostate cancer, financial toxicity, cost estimates, multidisciplinary collaboration, shared decision making (SDM), quality of life

## Abstract

**Background:**

Many individuals undergoing cancer treatment experience substantial financial hardship, often referred to as financial toxicity (FT). Those undergoing prostate cancer treatment may experience FT and its impact can exacerbate disparate health outcomes. Localized prostate cancer treatment options include: radiation, surgery, and/or active surveillance. Quality of life tradeoffs and costs differ between treatment options. In this project, our aim was to quantify direct healthcare costs to support patients and clinicians as they discuss prostate cancer treatment options. We provide the transparent steps to estimate healthcare costs associated with treatment for localized prostate cancer among the privately insured population using a large claims dataset.

**Methods:**

To quantify the costs associated with their prostate cancer treatment, we used data from the Truven Health Analytics MarketScan Commercial Claims and Encounters, including MarketScan Medicaid, and peer reviewed literature. Strategies to estimate costs included: (1) identifying the problem, (2) engaging a multidisciplinary team, (3) reviewing the literature and identifying the database, (4) identifying outcomes, (5) defining the cohort, and (6) designing the analytic plan. The costs consist of patient, clinician, and system/facility costs, at 1-year, 3-years, and 5-years following diagnosis.

**Results:**

We outline our specific strategies to estimate costs, including: defining complex research questions, defining the study population, defining initial prostate cancer treatment, linking facility and provider level related costs, and developing a shared understanding of definitions on our research team.

**Discussion and next steps:**

Analyses are underway. We plan to include these costs in a prostate cancer patient decision aid alongside other clinical tradeoffs.

## Introduction

“Financial toxicity” (FT) is the personal financial burden faced by those undergoing cancer treatment, specifically the harms associated with this burden (Yousuf, 2016). Any individual with cancer may experience FT, including those with prostate cancer. In the US, prostate cancer is the most commonly diagnosed cancer in men and the second leading cause of cancer-specific mortality (Siegel et al., 2022). For patients with localized prostate cancer, the type of treatment they choose contributes to their susceptibility to FT, with radiation and surgery often having greater direct costs, financial burden, and variability over time (Imber et al., 2020; Stone et al., 2021). Patients experiencing FT are more likely to report nonadherence to medication, inability to afford prescription drugs, and forgoing mental health services, doctor’s visits, and medical tests (Knight et al., 2018). FT is associated with disparate health outcomes and lower quality of life (Yousuf, 2016).

Survival is similar for non-metastatic, localized prostate cancer across treatment options (i.e., radiation, surgery, active surveillance), but patients must weigh quality of life tradeoffs (e.g., distress, urinary incontinence, erectile dysfunction) during this preference-sensitive decision (Bill-Axelson et al., 2014; Lamers et al., 2017; Sanda et al., 2018). Providing cost estimates of the cost burden associated with different prostate cancer treatment pathways alongside clinical tradeoffs can support this decision; cost can be a substantial quality-of-life tradeoff that is often not discussed or not precisely known to patients during decision-making (Politi et al., 2021). There is a growing call regarding the importance of including direct and indirect cost information in shared-decision making conversations for prostate cancer as nonmetastatic treatment outcomes are generally similar and costs can help inform patients as they weigh their options (Ubel et al., 2013; Politi et al., 2023). Direct costs include insurance related fees (e.g., co-pays, co-insurance) and indirect costs include the often unforeseen costs (e.g., loss of work, absenteeism, presenteeism). Even with interest from patients and clinicians, cost conversations can be difficult to navigate(Kelly et al., 2015) due to the multidimensional nature of costs, impacting material, behavioral, and psychosocial domains (Tucker-Seeley and Thorpe, 2019). Discussing cost burden with patients upfront can enable patients to consider potential tradeoffs, seek financial assistance early on in their care, and thus potentially reduce future costs and the burden of care (Ubel et al., 2013; George et al., 2021).

In this paper, we aimed to quantify direct care costs and the associated financial burden for patients aged 18–63 years diagnosed with localized prostate cancer as the first step. We plan to incorporate this data into shared decision making materials and support patients as they consider which treatment option is right for them. Cost information will help patients consider both side effects and financial burden when they make decisions about their treatment among different treatment options. In this paper, we outline the steps involved in estimating direct costs following a prostate cancer diagnosis using insurance claims data. This report outlines lessons learned and recommendations for other researchers conducting similar analyses.

## Methods

### Step 1: identifying the problem

This research question arose from an existing project, evaluating a prostate cancer treatment decision aid that includes relative cost information led by a member of the research team (Politi et al., 2021). Formative interviews identified a gap in cost information for those making decisions about prostate cancer treatment options and their clinicians. Clinicians wanted to know more about these costs and patients and caregivers wanted to share more about the impact of immediate and downstream direct and indirect costs on their life (Politi et al., n.d.). Consequently, our research question was informed by the clinical, research, community and patient partners engaged in this formative work. Our research team prioritized engaging with these partners throughout the duration of this project. We knew this complex problem would also require the expertise of a multidisciplinary team as it spans patient care, clinical decision making, patient-centered communication, and economic evaluation.

### Step 2: engaging a multidisciplinary team

To develop a multidisciplinary collaborative team, the team met to discuss the research question and identify a potential funding source prior to approaching other team members. We invited a community collaborator and leader of a prostate cancer community-based organization to join our core research team and engage with local and regional community partners to incorporate their perspectives on costs and their impact on patients. We also engaged an urologist with clinical expertise on prostate cancer treatment, a health economist with expertise in cost analyses using administrative claims data, and a community-engaged researcher and leader of a local cancer center. We identified the need for expertise in these specific disciplines because of the complexities of calculating costs incorporating the clinical, economic, and community perspectives. To ensure our questions were clinically relevant and our operational definitions were accurate, a practicing urologic surgeon scientist helped generate and review the treatment definitions, billing and procedure codes, and define the clinical context. The health economist with expertise in estimating patient direct costs and large claims databases has worked extensively with data scientists on the institutional informatics team to oversee the analyses. The community-engaged researchers with expertise in health disparities provided important perspectives on the disproportionate experiences of FT by those from socially, economically, and racially marginalized groups. A postdoctoral trainee with expertise in cancer survivorship to support focusing on the impact of cost upfront and through survivorship over time. With this team of content and research experts, we also identified a research coordinator with extensive experience in clinical decision support informed by billing and procedure codes to oversee the administrative aspects of this project.

### Step 3: reviewing the literature and identifying the database

Through engaging our multidisciplinary team, we identified, reviewed, and selected the codes to extract, with this process occurring over multiple phases. First, the research team reviewed existing literature to identify procedure and billing codes. This involved reviewing peer reviewed literature and guidelines. Second, we reviewed the procedure codes included in the Fair Health Consumer^1^ prostate cancer shared decision making cost tool. Third, we reviewed the National Library of Medicine’s Value Set Authority Center (VSAC; https://vsac.nlm.nih.gov/) and the Unified Medical Language System (UMLS; https://www.nlm.nih.gov/research/umls/index.html). The research team compiled these resources, reviewed them together, and confirmed the procedure set we would use in this project. Our clinical team member, a practicing urologic surgeon, led iterative review and selection of procedure codes and discussed with clinical partners, including a radiation oncologist with experience working in claims data, when there were uncertainties about which to include. Our final code set is included in Table 1.

**Table 1:** Summarized list of treatment options, procedures, and CPT codes for the 3 treatment types.*

Treatment option	Procedure	CPT codes
Active surveillance	Biopsies	55700–55706
	Pelvic MRI	72195–72197
Prostatectomy	Open	55840
	Laparoscopic	55866
Radiation	Temporary hormones	J9218, J9202, J3315, J3489, J0897
	External beam	77401–77416 and G6003-G6014
	Seeds/internal/brachytherapy	77263
	Fiducial marker placement	55876
	Biodegradable injections	55874

* Please see the supplementary information for the complete list of CPT codes used in this analysis.

Based on this review, our research team decided to use data from the Truven Health Analytics MarketScan Commercial Claims and Encounters, including MarketScan Medicaid, (MarketScan). We selected MarketScan because of the inclusion of variables needed for our research question and to conduct analyses, national representativeness of a privately insured population, extant literature using MarketScan for similar analyses on financial burden in cancer survivors, and the availability and expertise within our institution. While the median age for prostate cancer is 66 years, over 170 million people under 65 years are covered by private health insurance (National Health Statistics Reports, 2021). Specifically, there were 224,733 new prostate cancer cases diagnosed in the US in 2019, and 37% of those cases were among men aged 45–64 years (Prostate Cancer Incidence by Stage at Diagnosis, 2023). These cost estimates will be relevant to this large group of people. Individuals with private insurance often spend more on care, have more medical debt, and report that costs impact care access (Wray et al., 2021). Those under 65 years are often exposed to more variable costs and cost estimates may be particularly relevant to this population.

### Step 4: identifying outcomes

Based on the findings from our initial work and literature review, the research team identified that treatment-related costs can occur over time, and a single time point would be unable to capture the costs across a trajectory of prostate cancer care. Thus, we quantified these costs cumulatively at 1 year, 3 years, and 5 years. Estimating costs at multiple time points would provide a better estimate of patient costs over time (Eldefrawy et al., 2013; Gustavsen et al., 2020). Using the MarketScan database for data extraction, we created an analyzable dataset to estimate the patient, clinician, and system/facility costs. Initially, our goal was to estimate these costs for patients with localized prostate cancer. Ideally, localized prostate cancer would be defined by Gleason, PSA, or tumor staging data, but these variables are not available in the MarketScan, despite the many strengths that prompted us to choose to use this database. Considering this limitation, we chose to use the metastatic vs. non-metastatic variable to define our cohort of interest.

### Step 5: defining the cohort

**Figure 1:**
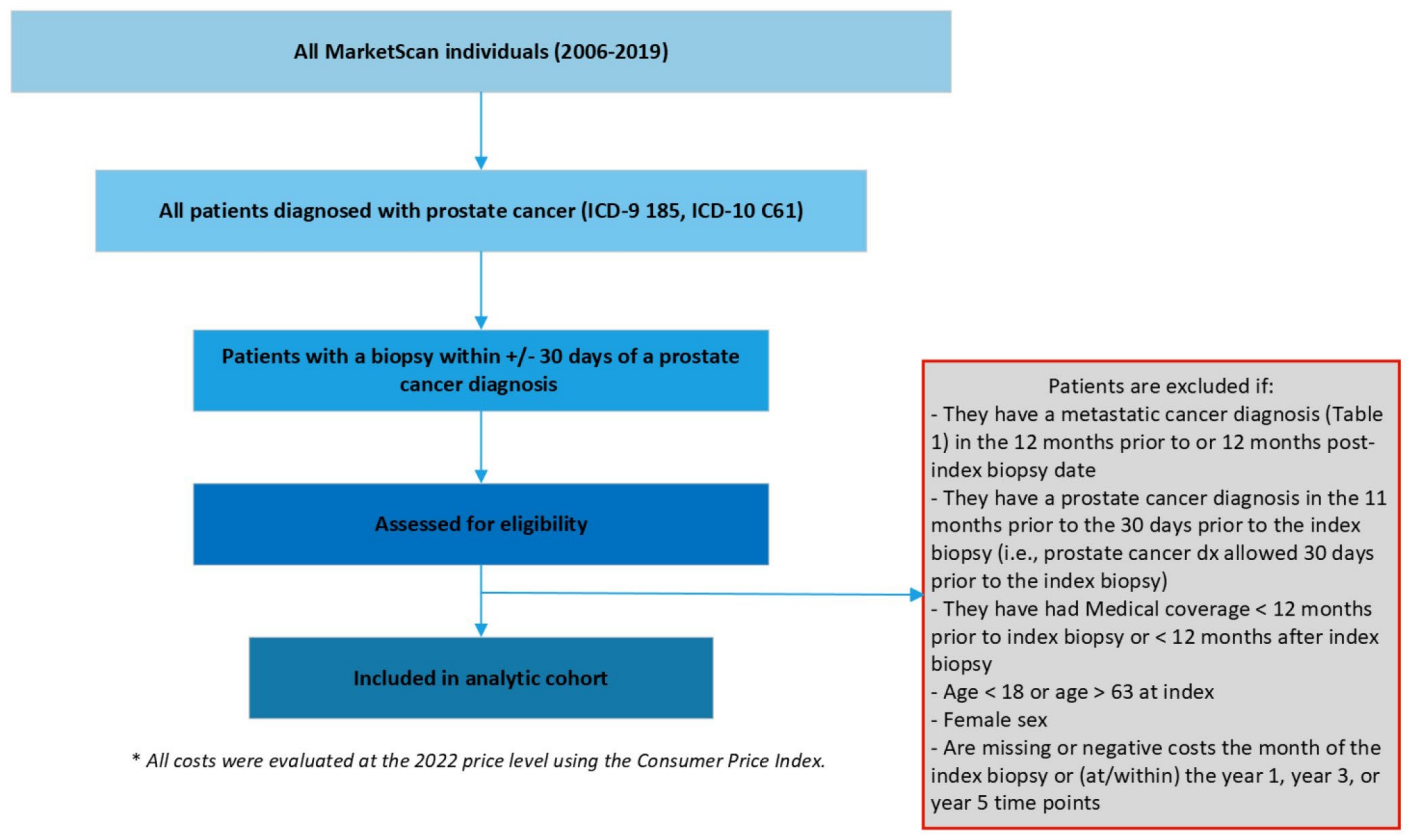
Defining the analytic cohort.

We defined localized prostate cancer as being diagnosed with prostate cancer and the absence of metastatic diseases using International Classification of Diseases, Ninth/Tenth Revision, Clinical Modification (ICD-9/10-CM). We first included patients with at least 2 outpatient codes at least 30-days apart or one inpatient prostate cancer diagnosis (ICD-9185, ICD-10 C61) between 2006 and 2019 (the most updated data at the time of study). Among these patients, the date of diagnosis (index date hereafter) was defined as the date of the first biopsy within +/− 30 days of a prostate cancer diagnosis, as biopsy is needed to determine a diagnosis and the dates of biopsy and diagnosis may lag administratively. Patients without a date of diagnosis were excluded. Additional exclusion criterion included patients: (1) with a secondary cancer diagnosis other than prostate cancer, (2) with a metastatic cancer diagnosis in the 12-months prior to or post the index date, (3) with a prostate cancer diagnosis in the 11-months prior to the index date, (4) with medical coverage <12-months prior to index date or < X-year after index date since this indicates incomplete cost data, where X = 1, 3, or 5 (i.e., the duration of the target cumulative cost of interest), (5) age < 18 years or age > 63 years at index (for the concern of incomplete data due to Medicare eligibility), (6) female sex, and (7) missing or negative costs within the duration of the target cost due to administrative data entry errors (see Figure 1).

#### Defining the treatment groups

We defined a patient’s initial treatment decision as the first treatment codes present within the 12-months following their index biopsy code. We categorized patients into 3 groups based on their initial treatment choice following diagnosis: active surveillance, surgery, and/or radiation using the Current Procedural Terminology (CPT) codes (see Table 1). Surgery included laparoscopic and open prostatectomy. Radiation included external beam and seeds/internal/brachytherapy. Active surveillance was defined as having no surgery or radiation codes within 12-months of the index date. Specifically, within 12-months following diagnosis, if a patient did not have treatment codes for either surgery or radiation, the patient was considered to have selected active surveillance. We estimated the cost associated with this treatment and all follow-up costs within the 5-year period, including other potential treatments (i.e., surgery, radiation). This approach captures all treatment related costs associated with their initial treatment decision.

### Step 6: designing the analytic plan

The analytic plan was finalized as the research team refined our research question and defined our variables. The analytic plan was an iterative process and refined as the team identified the data available in the database, the variables of interest, and our overall research objectives. Our team decided we would quantify total costs at 1, 3, 5 years following diagnosis and aggregate these costs across those time points. Total costs were evaluated from the healthcare sector’s perspective, including patients’ out-of-pocket cost and cost paid by third party payers. Costs pooling from different years were evaluated at the 2022 price level using the Consumer Price Index Medical Care Component (Consumer Price Index (CPI), 2023). For unadjusted analysis, we plan to average costs across patients receiving each treatment option to calculate mean costs for each treatment pathway. For adjusted analyses, the distribution of the total costs at different years will be visually examined. Appropriate statistical analyses will be determined and performed using these total costs as the outcome variables with covariates including patient characteristics (e.g., age, comorbidities), insurance types, geographical region where they received treatments, and treatments that they received. Total costs by initial treatment decision will be predicted based on the estimated regression at the three time periods based on patient characteristics, insurance type, geographical region, and a combination of treatment options.

## Results

### Lessons learned and strategies

A summary of lessons learned, strategies, and examples is described below in detail and summarized in Table 2.

**Table 2:** Challenges and recommendations.

Challenge	Recommendation	Example solution
Identifying the research expertise needed and ensuring shared understanding across multiple disciplines and progress updates.	Leverage the range of expertise by engaging a multiple disciplinary team from clinical, scientific, and community-based perspectives. At the beginning of the project, prioritize dedicating time to selecting team members that represent expertise in the priority areas for your project. Their perspectives and knowledge are essential for identifying the relevant treatment codes, reviewing the codes to ensure they make sense clinically, and confirming the data are available within the dataset, conducting the analysis, and interpreting the results.	Our team is made up of experts in oncology, urology, public health, economics, data science, decision science, psychology, occupational therapy, and community engaged research. We conducted twice weekly, 30-min meetings and circulated detailed meeting minutes to clarify disciplinary jargon and ensure mutual understand of our approach and plan.
Defining the cohort for non-metastatic PCa without Gleason, PSA, or tumor staging data (limitations to the data set).	Review the variables available in the dataset and their clinical meaning to identify a strategy for how to address the research question. Determine how to use the available variables to define your cohort, relevant costs, and treatment pathways as accurately as possible. This can include using specific billing codes, procedure codes, time intervals, absence or presence of codes, among other strategies.	In the absence of available variables for Gleason, PSA, and tumor staging data, we used metastatic vs. non-metastatic cancer diagnosis to help define a patient cohort with localized prostate cancer. This distinction comes with additional considerations, including how to identify the non-metastatic cohort and when to exclude metastatic cases (e.g., upfront, at a certain period of time). Our team defined non-metastatic as: (1) a diagnosis with prostate cancer and no metastatic cancer codes present at any point prior to and/or 30 days after the index date and (2) patients who progress to metastatic prostate cancer within 12-months after the index date will be excluded; however patients progressed after 12-months will be included.
Identifying procedure and billing codes.	Review existing literature, cross-reference resources for ICD-9/10 and CPT code identification including the National Library of Medicine’s Value Set Authority Center (VSAC) and the Unified Medical Language System (UMLS). This is an iterative process that requires repeated review and confirmation from key team members (i.e., clinical expert).	We compiled a list of ICD-9/10 and CPT through: (1) peer reviewed literature and guidelines, (2) reviewed procedure codes from existing patient facing resources (i.e., Fair Health Consumer), and (3) national repositories (VSAC; UMLS). The research team, including a clinical expert, complied these resources, reviewed them together, and confirmed the final procedure set.
Identifying when a patient chooses active surveillance	Active surveillance is not a specific CPT code and therefore requires applying a definition for how active surveillance will be operationalized. This can be done by reviewing extant literature, discussing with a multidisciplinary team, reviewing medical system processes and procedures, and defining treatment option.	Since the absence of surgery or radiation codes does not necessarily indicate an active surveillance treatment choice, the team worked together through multiple iterations of our operational definition for active surveillance. Based on a literature review, the team’s knowledge of medical system processes, and consultation with a urologist routinely involved in patient care and billing, active surveillance was defined as: patients diagnosed with prostate cancer who, within 12-months of diagnosis, have billing codes for biopsy or biopsy and pelvic MRI an no other treatment codes.
Linking facility and provider level related costs using treatment variables.	By using inpatient facility claims, only CPT codes will be used to identify the procedures and there is a discrepancy between the number of people with facility costs vs. the number with provider costs.	Link facility claims +/− 1 day to provider surgery claims. We are doing this for inpatient facility claims as well as outpatient facility claims to capture any procedures that were done on an ambulatory basis.
Shared understanding of definitions and documentation of research questions	On our team of experts, we needed to closely manage and document our operational definitions and current status of the project. At each key decision point, find a way to confirm the approach with team members and receive individual and group approval. This will eliminate confusion and facilitate effective collaboration.	Brief, frequent team meetings; shared box folder; sub-group working meetings and circulating minutes with the full team; project dictionary for key terminology to document operational definitions; regular email updates outlining most-recent updates and key decisions.

### Complex research questions across multiple disciplines

Our team met frequently and worked together to translate our research questions across discipline specific language, including across oncology, urology, public health, economics, data science, decision science, psychology, occupational therapy, and community engaged research. Initially our meetings were 60-min every other week, but we increased the frequency to meeting for 30-min twice a week. While this increased frequency can be demanding to the research team members, we found as the momentum of our project started to increase, we needed rapid feedback and to update the team on progress. We did cancel meetings if they were not needed and corresponded over email to update the team. We also shared detailed meeting minutes to keep all team members apprised of updates. Through our frequent, brief meetings, we refined our analytic plan, and we were able to ask questions in real time to address and translate discipline specific jargon and assumptions, and ultimately agree on our analytic process. We then created a draft analysis plan to circulate with the research team to elicit additional input from our team members. Through this process, we incorporated scientific, medical, and community perspectives to refine our questions and define a clinically meaningful cohort within the larger dataset. This is an ongoing process as new information emerges or challenges arise, yet our goal is to identify these issues early and often so we can address them in a way that aligns with the research question, data, science, clinical relevance, and patient experiences.

### Defining the cohort

Due to the aforementioned limitations of unavailable data to define localized prostate cancer in the MarketScan database, we worked with our team to identify which metrics/measures exist and how to feasibly extract them from the database. Through this process, our team elected to use metastatic vs. non-metastatic cancer diagnosis to help define localized prostate cancer. This distinction comes with additional considerations, including how to identify the non-metastatic cohort and when to exclude metastatic cases (e.g., upfront, at a certain period of time). We selected the time parameters to provide the framework needed to verify the confirmed prostate cancer records. Primarily, we determined that the date of a patient’s initial biopsy would serve as the index date. 12-months before the prostate cancer index date no ICD codes for prostate cancer beyond 30-days from the index date and if both biopsy and diagnostic codes are present within 1-month, we will consider this patient to be diagnosed with prostate cancer. Limitations to this approach include those patients who may have had a biopsy outside of what was captured in the MarketScan database, but the research team evaluated the tradeoffs between a smaller sample size and a well-defined patient cohort (patients diagnosed with non-metastatic prostate cancer) and erred on the side of caution to include confirmed prostate cancer records in our analysis.

### Identifying procedure, billing codes, and patient treatment decision making

Our team reviewed existing databases to cross-reference resources for ICD-9/10 and CPT code identification. This included FairHealth, and the National Library of Medicine’s Value Set Authority Center (VSAC) and the Unified Medical Language System (UMLS). Specifically, we needed to define what the operational definition would be for active surveillance as it is the absence of a discrete treatment event, rather a cluster of treatment events over time. At what time point can we determine the patient has chosen active surveillance? Our research team defined selecting active surveillance as a prostate cancer diagnosis and the absence of surgery or radiation related codes within the 12-months post index diagnosis (i.e., first biopsy). Our team decided this timeframe was a clinically meaningful timeframe in which you would expect a patient to initiate and commence their initial treatment plan.

### Defining treatment decisions

For those categorized as selecting active surveillance, if the patient transitioned to another treatment type as defined by the presence of treatment-related codes (i.e., surgery, radiation; Table 1) after 12-months, we included these patient records since we are interested in capturing all treatment related costs associated with their initial treatment decision. This approach allowed us to estimate the overall costs (initial costs and follow-up costs) associated with following treatment paths: (1) first electing active surveillance treatment (at 1-year, 3-years, and 5-years), (2) first electing surgery treatment (at 1-year, 3-years, and 5-years), and (3) first electing radiation treatment (at 1-year, 3-years, and 5-years).

### Linking facility and provider level related costs using treatment variables

Using CPT codes to identify the procedures lead to a discrepancy between the number of people with facility costs versus the number with provider costs. To avoid a systematic missing of facility costs, our research team considered: (1) either providing the ICD-9/10 procedure codes for treatments that would logically be done during an inpatient admission (especially the surgical procedures) or (2) linking facility claims to provider surgery claims based on dates (+/− 1 day). We decided to use approach 2 because each case would likely vary and approach 2 would be more inclusive of all associated costs.

### Shared understanding of definitions and documentation of research questions

One of our team’s main challenges has been reaching a shared understanding of the definitions and criteria for our analytic plan. We have adopted several strategies to help enhance communication and achieve consensus among our multi-disciplinary team. Primarily, we have conducted frequent, short meetings to ensure that all team members are updated and to create space to troubleshoot issues and misconceptions. In addition, we have created a centralized location for all files and realized the importance of regularly updating documents and operational definitions that are iteratively adjusted. We have also identified the importance of sending team-wide email updates after any modification is made to the analytic plan, cohort definitions, or inclusion and exclusion criteria. Not all team members are able to be present at each meeting, so regular email updates have also been a critical method for communicating changes and maintaining consensus.

### Discussion and next steps

Cost analyses are currently underway. We will be estimating total costs at 1-year, 3-years, and 5-years following diagnosis. In parallel, we are conducting semi-structured interviews among Black prostate cancer survivors and their caregivers to characterize the role of direct and indirect costs during their prostate cancer treatment through lived experiences. We are planning to include these direct and indirect costs in a prostate cancer patient decision aid and test this decision aid among patients with localized prostate cancer.

### Limitations

This research approach is not without limitations. First, active surveillance and watchful waiting are very different treatment types philosophically and in practice, but it is challenging to differentiate these approaches using claims data as the billing records may appear to be the same. Therefore, our multidisciplinary research team agreed upon using 12-months as the timeframe to suggest a patients’ selection of active surveillance. However, this could include some watchful waiting patients, which has the potential to artificially lower cost estimates. Additionally, MarketScan data does not include data on PSA, Gleason score, or tumor staging data. While this limited our ability to define low risk prostate cancer, the overall goal of the analysis is to better understand treatment-specific costs. Selecting a prostate cancer treatment pathway is a preference-sensitive decision, and therefore it is still important to include this information. MarketScan only includes claims data for those who are insured (including those eligible for Medicaid and Medicare), precluding those who are uninsured from our analysis. Together with a lack of race or ethnicity data, our analysis is not able to consider health equity. To address this, our larger research project includes a second aim where we will conduct qualitative interviews with Black men with prostate cancer to identify and further explore the direct and indirect costs associated with their treatment. We are also continually learning new information about our approach and analysis and identifying challenges. With new information and challenges, we will make informed decisions for how to proceed with the input of our team. Thus, our final analytic plan will be reported at the end of this research project. Our goal for this paper is to rapidly translate our methods and strategies to other researchers grappling with similar questions in an effort to facilitate academic discourse and increase transparency.

### Conclusions

Leveraging the expertise of a multidisciplinary team can help to identify the essential factors needed to estimate patient-related costs. These are complex research questions that evolve iteratively as additional information is uncovered through identifying the variables and clarifying the analytic plan. As we finalize our decision aid with cost information from this work, we will engage with clinical, patient, caregiver, community and decision science partners to review the presentation of information and identify supports needed to implement in routine care. We will prioritize recruiting from socially and economically marginalized populations to evaluate how the inclusion of costs may support decision making because of the disproportionate financial burden experiences by these populations.

## Data availability statement

The original contributions presented in the study are included in the article/Supplementary material, further inquiries can be directed to the corresponding author.

## Author contributions

AH: conceptualization, methodology, investigation, writing (original draft, review, editing), resources, supervision, and funding acquisition. HR: investigation, writing (original draft, review, editing), and visualization. S-HC: conceptualization, methodology, investigation, writing (review, editing), supervision, and funding acquisition. AL’H: investigation, writing (review, editing). EK and MP: conceptualization, methodology, writing (review, editing), and funding acquisition. BD and RW-J: conceptualization, writing (review, editing), and funding acquisition. All authors contributed to the article and approved the submitted version.

## Funding

This project was funded, in part, by The Implementation Science Centers in Cancer Control (ISC3; Beau Biden Cancer Moonshot Initiative, NCI P50 CA244431), Washington University and Siteman Cancer Center Institutional matching funds, and formative research was funded by Robert Wood Johnson Foundation grant number 77292.

## Conflict of interest

RW-J was employed by The Empowerment Network Inc., United States. MP was a consultant for UCB Biopharma in 2022 on a topic unrelated to this manuscript.

The remaining authors declare that the research was conducted in the absence of any commercial or financial relationships that could be construed as a potential conflict of interest.

## Publisher’s note

All claims expressed in this article are solely those of the authors and do not necessarily represent those of their affiliated organizations, or those of the publisher, the editors and the reviewers. Any product that may be evaluated in this article, or claim that may be made by its manufacturer, is not guaranteed or endorsed by the publisher.

## Author disclaimer

The contents of this paper are solely the responsibility of the authors and do not necessarily represent the official views of the National Institutes of Health or other funding agencies.

## Footnotes

^1^ https://www.fairhealthconsumer.org/

## References

[ref1] Bill-Axelson A. Holmberg L. Garmo H. Rider J. R. Taari K. Busch C.. (2014). Radical prostatectomy or watchful waiting in early prostate Cancer. N. Engl. J. Med. 370, 932–942. doi: 10.1056/NEJMoa1311593, PMID: 24597866 PMC4118145

[ref2] Consumer Price Index (CPI). Databases: U.S. Bureau of Labor Statistics (2023). Available at: https://www.bls.gov/cpi/data.htm

[ref3] Eldefrawy A. Katkoori D. Abramowitz M. Soloway M. S. Manoharan M. (2013). Active surveillance vs. treatment for low-risk prostate cancer: a cost comparison. Urol. Oncol. 31, 576–580. doi: 10.1016/j.urolonc.2011.04.005, PMID: 21616691

[ref4] George N. Grant R. James A. Mir N. Politi M. C. (2021). Burden associated with selecting and using health insurance to manage care costs: results of a qualitative study of nonelderly Cancer survivors. Med Care Res Rev MCRR. 78, 48–56. doi: 10.1177/1077558718820232, PMID: 30569838

[ref5] Gustavsen G. Gullet L. Cole D. Lewine N. Bishoff J. T. (2020). Economic burden of illness associated with localized prostate cancer in the United States. Future Oncol Lond Engl. 16, 4265–4277. doi: 10.2217/fon-2019-0639, PMID: 31802704

[ref6] Imber B. S. Varghese M. Ehdaie B. Gorovets D. (2020). Financial toxicity associated with treatment of localized prostate cancer. Nat. Rev. Urol. 17, 28–40. doi: 10.1038/s41585-019-0258-3, PMID: 31792431 PMC8010900

[ref7] Kelly R. J. Forde P. M. Elnahal S. M. Forastiere A. A. Rosner G. L. Smith T. J. (2015). Patients and physicians can discuss costs of Cancer treatment in the clinic. J. Oncol. Pract. 11, 308–312. doi: 10.1200/JOP.2015.003780, PMID: 26015459 PMC4507390

[ref8] Knight T. G. Deal A. M. Dusetzina S. B. Muss H. B. Choi S. K. Bensen J. T.. (2018). Financial toxicity in adults with Cancer: adverse outcomes and noncompliance. J. Oncol. Pract. 14, e665–e673. doi: 10.1200/JOP.18.00120, PMID: 30355027

[ref9] Lamers R. E. D. Cuypers M. de Vries M. van de Poll-Franse L. V. Ruud Bosch J. L. H. Kil P. J. M. (2017). How do patients choose between active surveillance, radical prostatectomy, and radiotherapy? The effect of a preference-sensitive decision aid on treatment decision making for localized prostate cancer. Urol Oncol Semin Orig Investig. 35, 37.e9–37.e17. doi: 10.1016/j.urolonc.2016.09.007 28341494

[ref10] National Health Statistics Reports (2021). Number 159, 2021;(159). Available at: https://www.cdc.gov/nchs/data/nhsr/nhsr159-508.pdf

[ref11] Politi M. C. Forcino R. C. Parrish K. Durand M. A. O’Malley A. J. Elwyn G. (2021). Cost talk: protocol for a stepped-wedge cluster randomized trial of an intervention helping patients and urologic surgeons discuss costs of care for slow-growing prostate cancer during shared decision-making. Trials 22:422. doi: 10.1186/s13063-021-05369-4 34187547 PMC8240421

[ref12] Politi M. C. Forcino R. C. Parrish K. Durand M. A. O’Malley A. J. Moses R.. (n.d.). The impact of adding cost information to a conversation aid to support shared decision making about low-risk prostate cancer treatment: results of a stepped-wedge cluster randomized trial. Health Expect. 10.1111/hex.13810 PMC10485319 37394739

[ref13] Politi M. C. Housten A. J. Forcino R. C. Jansen J. Elwyn G. (2023). Discussing cost and value in patient decision aids and shared decision making: a call to action. MDM Policy Pract. 8:238146832211486. doi: 10.1177/23814683221148651 PMC9834940 36643615

[ref14] Prostate Cancer Incidence by Stage at Diagnosis. United States—2001−2019 | CDC [Internet] (2023) cited 2023 May 31. Available at: https://www.cdc.gov/cancer/uscs/about/data-briefs/no34-prostate-cancer-incidence-2001-2019.htm

[ref15] Sanda M. G. Cadeddu J. A. Kirkby E. Chen R. C. Crispino T. Fontanarosa J.. (2018). Clinically localized prostate Cancer: AUA/ASTRO/SUO guideline. Part II: recommended approaches and details of specific care options. J. Urol. 199, 990–997. doi: 10.1016/j.juro.2018.01.002, PMID: 29331546

[ref16] Siegel R. L. Miller K. D. Fuchs H. E. Jemal A. (2022). Cancer statistics, 2022. CA Cancer J. Clin. 72, 7–33. doi: 10.3322/caac.21708, PMID: 35020204

[ref17] Stone B. V. Laviana A. A. Luckenbaugh A. N. Huang L. C. Zhao Z. Koyama T.. (2021). Patient-reported financial toxicity associated with contemporary treatment for localized prostate Cancer. J. Urol. 205, 761–768. doi: 10.1097/JU.0000000000001423, PMID: 33252300

[ref18] Tucker-Seeley R. D. Thorpe R. J. (2019). Material-psychosocial-behavioral aspects of financial hardship: a conceptual model for Cancer prevention. The Gerontologist 59, S88–S93. doi: 10.1093/geront/gnz033, PMID: 31100144 PMC6524757

[ref19] Ubel P. A. Abernethy A. P. Zafar S. Y. (2013). Full disclosure — out-of-pocket costs as side effects. N. Engl. J. Med. 369, 1484–1486. doi: 10.1056/NEJMp1306826, PMID: 24131175

[ref20] Wray C. M. Khare M. Keyhani S. (2021). Access to care, cost of care, and satisfaction with care among adults with private and public health insurance in the US. JAMA Netw. Open 4:e2110275. doi: 10.1001/jamanetworkopen.2021.10275, PMID: 34061204 PMC8170543

[ref21] Yousuf Z. S. (2016). Financial toxicity of Cancer care: It’s time to intervene. J. Natl. Cancer Inst. 108:djv370. doi: 10.1093/jnci/djv370 26657334

