# Supplementary material for: Financial burden of men with localized prostate cancer: a process paper
Source: Front Psychol. 2023 Jul 5;14:1176843. doi: 10.3389/fpsyg.2023.1176843 (PMC10354547; doi:10.3389/fpsyg.2023.1176843)
Supplement: Supplementary file 1 [file Table_1.pdf]

Supplementary Table 1.

**Initial CPT Codes Identified for Analysis.***		
**Treatment Type**	**Procedures**	**CPT Codes**
Active surveillance	Biopsies	55700-55706, 88305
	Pelvic MRI	72195-72197
Prostatectomy	Open	55840, 0370, 0300, 0310 0302, 0270, 0272, 0360, 0250, 0259, 0324, 0170, 0120, 55845, 99239, 99222, 86900, 86850, J1100, J0690, 88342, 88331, 88309
	Laparoscopic	558660730, 0301, 0305, 0312, 0271, 0278, 0258, 0320, 0121, 00865, 99232, 99223, J3010, J2704, J2250, J1170, 86901, 85027, 80048, J7120, J1644, 38571
Radiation	Temporary hormones	J9218, J9202, J3315, J3489, J0897
	External beam	77401-77416, G6003-G6014, 77385, 96402, 77014, G0463, 99214, 99213, 85025, 84403, 84153, 80053, 36415, 81003, 81001, J9217, 77427, 77338, 77336, 77031, 77300, 77280, G6002
	Seeds/internal/brachytherapy	77263, 55875, 00400, J0131, C2639, C1717, C1715, J2405, 77778, 77370, 77318, 77295, 76965, 76873 , 76872
*Note: These codes may be updated as this research project is ongoing.		
